# Molecular Changes Underlying Genistein Treatment of Wound Healing: A Review

**DOI:** 10.3390/cimb43010011

**Published:** 2021-05-17

**Authors:** Matúš Čoma, Veronika Lachová, Petra Mitrengová, Peter Gál

**Affiliations:** 1 Department of Pharmacology, Faculty of Medicine, Pavol Jozef Šafárik University, 040 11 Košice, Slovakia; matus.coma@upjs.sk; 2 Department of Biomedical Research, East-Slovak Institute of Cardiovascular Diseases, Inc., 040 11 Košice, Slovakia; 3 Department of Pharmacognosy and Botany, Faculty of Pharmacy, Comenius University, 832 32 Bratislava, Slovakia; lachova5@uniba.sk (V.L.); petra.mitrengova@uniba.sk (P.M.); 4 Laboratory of Cell Interactions, Faculty of Medicine, Pavol Jozef Šafárik University, 040 11 Košice, Slovakia; 5 Prague Burn Center, Third Faculty of Medicine, Charles University, 100 34 Prague, Czech Republic

**Keywords:** skin wound, repair, regeneration, phytoestrogen, SERM, isoflavone, scar

## Abstract

Estrogen deprivation is one of the major factors responsible for many age-related processes including poor wound healing in postmenopausal women. However, the reported side-effects of estrogen replacement therapy (ERT) have precluded broad clinical administration. Therefore, selective estrogen receptor modulators (SERMs) have been developed to overcome the detrimental side effects of ERT on breast and/or uterine tissues. The use of natural products isolated from plants (e.g., soy) may represent a promising source of biologically active compounds (e.g., genistein) as efficient alternatives to conventional treatment. Genistein as natural SERM has the unique ability to selectively act as agonist or antagonist in a tissue-specific manner, i.e., it improves skin repair and simultaneously exerts anti-cancer and chemopreventive properties. Hence, we present here a wound healing phases-based review of the most studied naturally occurring SERM.

## 1. Introduction

Improved healthcare in developed countries has led to an increase in the size of the elderly population. Therefore, almost one third of a woman’s life occurs in the post-menopausal period. Estrogen deprivation is one of the major factors responsible for many age-related processes including poor wound healing in postmenopausal women [1]. Wound healing is a dynamic event running in four basic phases, i.e., hemostasis, inflammation, proliferation, and maturation/remodeling, which overlap with each other in a consecutive manner restoring the disrupted integrity of tissues/organs. Immediately following blood clotting inflammatory/immune cells enter the injury site to eliminate pathogens and remove necrotic tissue [2]. Fibroblasts located in the granulation tissue excrete and remodel extracellular matrix (ECM) proteins, whereas differentiated myofibroblasts contract the wound [3]. In addition, endothelial cells create new vessels and provide nutritional support for the newly formed tissue [4]. To create a mechanical barrier between the outer and inner environment, keratinocytes migrate across the wound (over the granulation tissue) restoring the integrity of the organism [5].

Previously, it was shown that women receiving estrogen replacement therapy (ERT) have a lower incidence of chronic wounds [6,7]. Moreover, improved skin wound healing was also observed following topical application of estrogen [8]. In this clinical study ERT was shown to significantly reduce wound size, increase collagen deposition, and improve wound stiffness. Further beneficial effects of ERT in elderly women involve reduced risk of bone fracture, colorectal cancer, and incidence of menopausal symptoms [9,10]. However, reported side-effects of estradiol substitution have precluded a common clinical introduction of ERT. Accordingly, selective estrogen receptor modulators (SERMs) were studied to overcome the detrimental side effects of ERT on breast and/or uterine tissues [11,12]. A renewed strategy directed towards the use of natural products isolated from plants (e.g., soy) might represent a promising source of biologically active compounds (e.g., genistein) as efficient alternatives to conventional treatment. Hence, we present here a systematical, wound healing phases-based, review of the most studied naturally occurring SERM genistein.

## 2. Methods—Literature Search Strategy

The effects of genistein on wound healing were searched on PubMed using the term “genistein wound healing” for relevant English-written papers (Available online: https://pubmed.ncbi.nlm.nih.gov/?term=genistein+wound+healing&sort=date, accessed on 31 March 2021), the search identified 84 articles. The present paper reviews the most relevant studies and includes the appropriate references.

## 3. (Phyto)Estrogen Signaling in Wound Healing and Skin Aging

Menopause-induced estrogen deficiency is accompanied by changes in skin morphology and physiology involving decrease in thickness, collagen, and water content as well as loss of elasticity and increase in fragility [13,14,15]. Lower estrogen levels associated with the menopause also result in impaired wound healing. In particular, the negative effect of the menopause on wound healing results from fragile skin that tears and bruises easily and is thus susceptible to trauma [16].

Phytoestrogens are non-steroidal compounds of plant origin structurally and functionally similar to human estrogen [17]. Depending on endogenous estrogen levels and estrogen receptors (ERs) expression, phytoestrogens may act (tissue dependent) as agonists and/or antagonists, thus presenting a subclass of SERMs [18]. Genistein (4′,5,7-trihydroxyisoflavone) is a naturally occurring phytoestrogen (commonly found in soy products including soy beans except for soy sauce [19]) and the most studied natural SERM. The most prevalent form of genistein is the biologically active genistein-7β-glucoside, which on digestion or fermentation leads to release of the aglycone form [20]. Although well absorbed in the small intestine, genistein has low oral bioavailability due to low water-solubility, extensive metabolism, and high expression level of efflux transporters, especially breast cancer resistance protein (BCRP) [21,22,23]. Structurally genistein resembles 17β-estradiol, mostly due to the similar position and distance between the OH groups, crucial for the binding ability of both phytoestrogens and estrogens towards ERs. However, genistein has a higher binding affinity for ER-β than ER-α resulting in functional diversity between both molecules [24]. ER-β is predominantly expressed in the epidermis, dermal fibroblasts, blood vessels, and hair follicles and represents the main mediator of estrogen action in the skin [25,26]. Pharmacological effects of genistein are mediated through competitive agonizing/antagonizing of ERs. Apart from genomic estrogen signaling, several non-ER effects of genistein have been described including activation of G protein-coupled receptor 30 (GPR30) [27] and inhibition of protein-tyrosine kinases (PTK) [28,29], topoisomerase II [30], platelet-derived growth factor (PDGF) [31], and epidermal growth factor (EGF)-induced c-Fos expression, as well as inhibition of diacylglycerol synthesis [32] and angiogenesis [33,34]. Additionally, genistein modulates various features of cancer including cell cycle, apoptosis, angiogenesis, and metastasis [35]. Intriguingly, activity of genistein revealed a concentration dependent pattern where higher concentrations inhibited the activity of PTK associated with the EGF receptor, while lower concentrations triggered estrogenic activity [36,37].

## 4. Inflammatory Phase and Oxidative Stress

**Figure 1:**
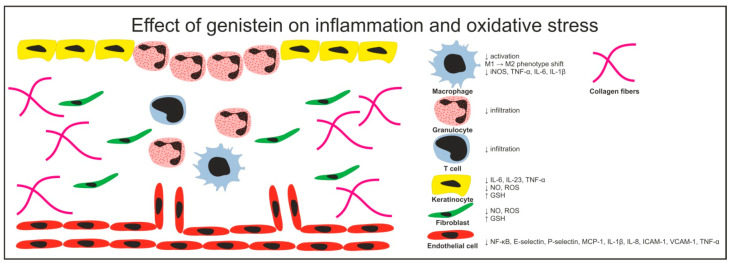
Cell-specific effects of genistein on wound healing during the inflammatory phase.

Inflammation is a crucial process involved in wound healing. During the inflammatory phase the innate immune response is activated leading to the recruitment of immune cells to the injury site and removal of invading pathogens and dead cells/tissues [38]. The imbalance between pro-inflammatory and anti-inflammatory signaling has a crucial influence on the morphological and functional outcome of scarring [39]. In particular, prolonged/chronic inflammation may cause deregulation of keratinocyte and fibroblast differentiation/activation which may in turn lead to disturbance in the wound healing process. frequently resulting in excessive scar formation [40]. Intriguingly, despite inappropriately excessive inflammatory response associated with age-related delayed wound healing [41], the menopause is rather associated with reduced scarring and ECM deposition [13,42]. The effect of genistein on inflammatory phase of wound healing and oxidative stress is shown in Figure 1.

In particular, macrophages are of high importance in wound healing as they attenuate inflammation, clear cell debris, as well as coordinate tissue repair. Genistein can inhibit activation of macrophages exposed to inflammatory stimulus by inhibiting activation of transcription factor nuclear factor kappa-light-chain-enhancer of activated B cells (NF-κB), as well as signal transducer and activator of transcription 1 (STAT-1) [43]. In detail, transcription factor NF-κB controls the expression of inducible chemokines, cell adhesion molecules, vasoactive proteins, and anti-apoptotic proteins important for cellular stress response. The NF-κB signaling pathway is triggered by inflammatory activators such as UV light, cytokines, free radicals, oxidized LDL (ox-LDL,) and viral antigens [44]. One part of the innate immune response regulated by NF-κB is NOD-like receptor protein-3 (NLRP3) inflammasome. NLRP3 inflammasome is reported to have an important role in promoting the early stage of skin wound healing [45]. Genistein supplementation had a positive effect on NLRP3 expression which was reduced by diabetes in mice [46]. Apart from in vitro data, the accelerated wound closure rate observed in the early stages of healing was accompanied by decreased levels of inflammatory markers TNF-α and NF-κB in genistein treated mice [46,47]. Furthermore, an in vivo study showed that genistein treatment can shift macrophage phenotype from M1 to M2 in rats with experimental colitis [48]. The switch from pro-inflammatory M1 phenotype to inflammation-resolving M2 phenotype in macrophages is crucial for the transition from the inflammatory to the proliferation phase of wound healing [40]. Genistein was also able to inhibit inducible nitric oxide synthase (iNOS) activity and production of cytokines, promoting inflammation such as tumor necrosis factor alpha (TNF-α), interleukin (IL)-6 and IL-1β in lipopolysaccharide (LPS)-stimulated macrophages [48].

Furthermore, genistein prevented LPS-induced decrease in adenosine monophosphate-activated protein kinase (AMPK) phosphorylation [49]. Of note, a novel molecule synthesized using genistein, 7-difluoromethoxy-5,4′-dimethoxy-genistein (DFMG), was able to attenuate macrophage activation induced by co-culture with lysophosphatidylcholine (LPC)-injured human umbilical vein endothelial cells (HUVE-12) cells, via inhibition of NF-κB through the signaling pathway involving toll like receptor 4 (TLR4) and myeloid differentiation factor 88 (MyD88) [50]. Impact of gamma irradiation, a common food preserving procedure, was also studied in soy bean compounds including genistein [51]. The experiment revealed that the bioactivity of genistein through gamma-irradiation does not increase macrophage cytotoxicity. Moreover, gamma-irradiated genistein exerts anti-inflammatory action through inhibition of mitogen-activated protein kinase (MAPK) and NF-κB signaling pathways [52].

Apart from macrophages, genistein can ameliorate the immune response of granulocytes and T cells in vivo. In detail, the cell-mediated immune response in ovariectomized (OVX) mice was tested by monitoring the delayed-type hypersensitivity (DTH). Treatment of OVX mice with 8–80 mg/kg genistein decreased DTH by 46–67%. Furthermore, histopathological examination revealed reduced cell-infiltration and lower numbers of CD4^+^ and CD8^+^ T cells in lymph nodes [53]. A similar suppressive effect on DTH was observed in non-OVX mice fed with genistein (30 mg/kg) where the granulocyte dependent inflammatory response was also ameliorated [54].

Moreover, genistein affected secretion of cytokines, growth factors, and other signaling molecules produced by non-inflammatory cells with considerable impact on the inflammation associated with the healing process. For example, in human umbilical vein endothelial cells (HUVECs), genistein attenuated the expression of pro-inflammatory mediators NF-κB, E-selectin, P-selectin, monocyte chemoattractant protein-1 (MCP-1), IL-8, Vascular cell adhesion protein 1 (VCAM-1), and Intercellular adhesion molecule 1 (ICAM-1) triggered by ox-LDL [55]. Pretreatment of HUVECs with genistein prior to exposition to inflammatory stimulus lowered the induced expression of pro-inflammatory mediators, such as TNF-α, IL-1β, MCP-1, IL-8, and ICAM-1 on both mRNA and protein levels [56]. A similar anti-inflammatory effect was observed in keratinocytes stimulated with TNF-α where genistein treatment resulted in the inhibition of NF-κB nuclear translocation and expression of inflammatory cytokines (TNF-α, IL-6 and IL-23) [57].

Genistein treatment improved pathological scores of cutaneous skin lesions in mice and impaired expression of proinflammatory factors IL-1β, IL-6, TNF-α, C-C motif chemokine ligand 2 (CCL2), IL-17, and IL-23 induced by imiquimod [57]. Notably, the anti-inflammatory effect of genistein may also be related to its antioxidative properties. It was shown that treatment of human keratinocytes and fibroblasts with genistein could prevent skin aging via modulation of glutathione (GSH) content and reactive oxygen species (ROS) release, endothelial/inducible (e/i)NOS dependent NO release, matrix metalloproteinases (MMPs) expression, and mitochondria membrane potential through mechanisms involving p38 MAPK, Akt and extracellular signal-regulated kinases 1/2 (ERK1/2) as downstream signaling associated with ERs and GPR30 [58], as well as by increasing superoxide dismutase (SOD) activity and B-cell lymphoma 2 (Bcl-2) expression in endothelial cells [59,60]. Importantly, as was shown in an in vivo study where OVX mice were co-treated with ER antagonist, the ameliorative effect of genistein on the inflammatory stage of wound healing seems to be independent of ER-mediated signaling. However, genistein treatment affected the next step of the healing process through ER-signaling as accelerated re-epithelization induced by genistein treatment was partially reversed by ER antagonist (ICI 182,780), but had little impact on the anti-inflammatory effect of genistein [61].

As shown in a mice model, genistein decreases expression of Cu/Zn-SOD and Mn-SOD to a low level, which was shown to be sufficient for coping with oxidative stress in the early stages of wound healing [47]. Furthermore, genistein was shown to be able to modulate the antioxidant defense system impaired by diabetic condition in mice and restored its function in the early stages of wound healing [46]. Additionally, genistein reduced oxidative stress in diabetic mice through suppression of iNOS and forkhead box O transcription factor 1 (FoxO1) activity. This resulted not only in alleviation of delayed wound healing but also in improved angiogenesis [62]. Protection against oxidative stress in skin was also observed in male rats fed with an isoflavone mixture of genistein and daidzein (2 or 20 mg/kg). The isoflavone mixture-treated group showed significantly greater thickness of the skin epidermis as well as higher amounts of collagen and elastic fibers in the dermis. Skin homogenates of the treated group showed a decrease in catalase activity and inhibited lipid peroxide formation in a dose dependent manner [63].

## 5. Proliferation Phase

**Figure 2:**
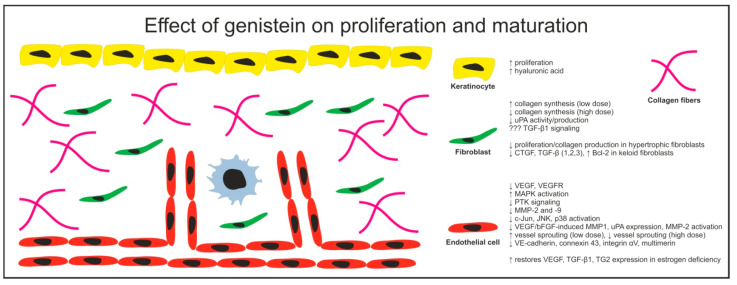
Cell-specific effects of genistein on wound healing during the proliferation and maturation phases.

The proliferative phase of wound repair involves formation of the granulation tissue including fibroblast proliferation, ECM deposition, angiogenesis, and wound re-epithelization [64]. In particular, several menopause-associated fibroblast malfunctions were reported in elderly women. The underlying mechanisms include microRNA-7 up-regulation, impaired function of EGF receptor, hyaluronan synthase 2, resulting in Januse kinase (JAK)/STAT1 over-activation [65]. In this context estrogen down-regulates microRNA-7 expression which attenuates STAT1 and also induces a rapid re-organization of the cytoskeleton in dermal fibroblast via the non-genomic GPR30 axis [66,67]. The effect of genistein on proliferation phase of wound healing is shown in Figure 2.

### 5.1. Fibroblasts

Dermal fibroblasts can transiently express α-smooth muscle actin (SMA) to obtain a myofibroblast-like phenotype [68]. Myofibroblasts play crucial role in ECM synthesis and remodeling, thus precise regulation of fibroblast-to-myofibroblast differentiation, e.g., with the main driving source molecule TGF-β1, is essential for proper wound closure [69]. Importantly, absence of initial TGF-β1 stimulus resulted in increased myofibroblast apoptosis. Interestingly, if initial TGF-β1 stimulus is exchanged to IL-10, the motility of fibroblasts increased and myofibroblasts number decreased [70]. Intriguingly, the crosstalk between ER and TGF-β1 signaling was reported [71,72]. In detail, ER signaling suppresses TGF-β1-induced activation of Sma and MAD-related protein 3 (SMAD3), whereas TGF-β1 signaling stimulates ER-mediated transcriptional activation [73]. From this point of view the question of whether genistein treatment interacts with TGF-β1-induced fibroblast-to-myofibroblast differentiation should be explored in further research.

Genistein was shown to be able to influence collagen production in human dermal fibroblasts under oxidative stress conditions induced by *t*-BHP (*t*-butylhydroperoxide). Oxidative stress diminished collagen synthesis in fibroblasts and treatment of cells with genistein exerted a biphasic effect on collagen expression. Low concentration (1 µM) restored the decreased collagen expression whereas high concentration (100 µM) enhanced the inhibitory action of *t*-BHP. Protective action of the lower tested genistein concentration (1 µM) was mediated via modulation of the insulin-like growth factor 1 (IGF-1) receptor expression and ERK1/2 associated pathway [74].

In addition, genistein may also improve treatment of non-healing leg ulcers, typical of increased proteolytic activity and strong expression of MMPs, urokinase-type plasminogen activator (uPA), and extracellular MMP inducer [75]. As shown in human gingival fibroblasts, genistein was able to inhibit basal uPA activity. Moreover, genistein was also able to inhibit EGF-stimulated uPA production. In detail, genistein inhibited phosphorylation of the EGF receptor following stimulation inhibiting EGF-related activation of JNK and ERK1/2 [76].

### 5.2. Angiogeneis

Angiogenesis is the formation of new blood capillaries from existing vasculature through sprouting. This process is crucial in physiological as well as pathological conditions e.g., wound repair, inflammation, invasion, and tumor metastasis [77]. In the process of wound repair, angiogenesis is essential for the delivery of oxygen and nutrients to the newly developed tissue. Angiogenesis is comprised of consecutive actions including migration and proliferation of endothelial cells orchestrated by growth factors, oxygen levels, and proteases [2]. Recently, it was revealed that genistein can impact the growth and budding of endothelial cells, the formation of new capillaries, and some of the signaling pathways connected to angiogenesis [78] also via alteration of vascular endothelial growth factor (VEGF). VEGF is one of the most important regulators of angiogenesis, of which inhibition leads to significant decrease in blood vessel formation [79]. Importantly, the effect of genistein on VEGF expression is dose and cell type dependent [33,80,81,82,83].

In HUVECs, genistein decreased both basal and hypoxia stimulated VEGF and VEGF receptor (VEGFR) expressions on both protein [83] and mRNA [84] levels. In addition, genistein was able to inhibit VEGF-induced HUVEC proliferation at 10 µM concentration [83]. In detail, genistein treatment prevents HUVEC activation by VEGF through activation of MAPK and inhibition of PTK signaling, resulting in decreased production and activity of MMP-2 and MMP-9 as well as decreased activation of c-Jun N-terminal kinase (JNK) and p38 [33]. Furthermore, cell pre-treatment with genistein blocks the VEGF/bFGF induced MMP-1 and uPA expression and activation of MMP-2 through modulation of their inhibitor expression TIMP-1, TIMP-2, plasminogen activator inhibitor 1 (PAI-1), endostatin, angiostatin, and thrombospondin [85,86]. Of note, MMPs support angiogenesis by degrading the components of ECM, promoting endothelial cell invasion and sprouting [87].

However, genistein treatment of HUVECs showed a bivalent dose-dependent effect on in vitro endothelial tube formation. At low concentration (0.001–1 µM), genistein was able to stimulate tube-like structure sprouting, while in contrast at higher tested concentration (25–100 µM) genistein hindered angiogenesis [88]. Interestingly, treatment with 1 µM genistein averted the loss of tubule network impaired by high glucose concentration, whereas lower genistein concentration (0.1 µM) did not manifest such an effect [62].

**Figure 3:**
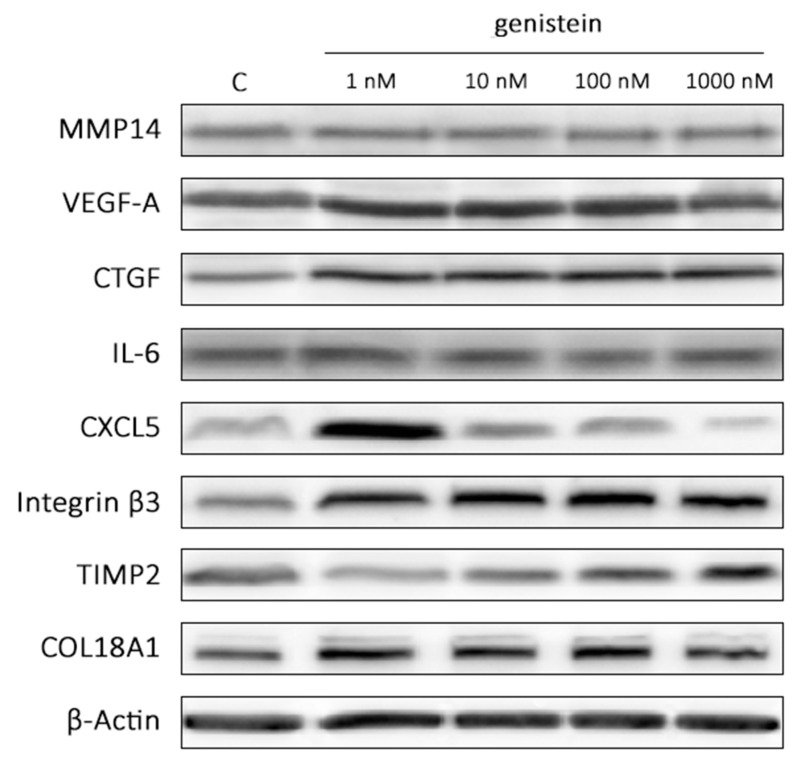
Western blot analysis of selected proteins performed on human microvascular endothelial cells (HMVEC-d) co-treated with VEGF (25 ng/mL) and increasing concentrations of genistein (1–1000 nM) after 48 h of incubation (MMP14—matrix metalloproteinase 14; VEGF-A—vascular endothelial growth factor A; CTGF—connective tissue growth factor; CXCL5—C-X-C motif chemokine 5; IL-6—interleukin 6; ITGB3—integrin β3; COL18A1—Collagen Type XVIII Alpha 1 Chain; TIMP-2—Tissue inhibitor of metalloproteinases 2).

Furthermore, genistein modulated angiogenesis by alteration of proangiogenic cytokines and protease expressions. In low doses (0.01–50 µM), genistein was shown to exert a positive effect on the secretion of bFGF, EGF, angiogenin, angiopoietin-2, MMP-9, and uPA receptor in HUVECs [88]. Interestingly, co-treatment of endothelial cells with VEGF and genistein at low concentrations (100 nM) showed a synergic effect on the up-regulation of six angiogenesis promoting genes—*MMP14*, *VEGF-A*, *CTGF*, *C-X-C motif chemokine 5* (*CXCL5*), *IL-6* and *integrin β3* (*ITGB3*)—as well as three angiogenesis inhibiting gene expressions—*Collagen Type XVIII Alpha 1 Chain* (*COL18A1*), *Tissue inhibitor of metalloproteinases* (*TIMP*)*-2*, and *TIMP-3* [59]. Therefore, we also used Western blot to confirm changes in gene expressions on the protein level. Our experiment revealed that out of nine dysregulated genes on the mRNA level, six (VEGF, CTGF, CXCL5, Integrin β3, TIMP2 and COL18A; TIMP3 not performed) were deregulated also on the protein level (Figure 3).

Of note, comparison of wound biopsies from OVX and sham operated rats showed a decrease in TGF-β1, VEGF, MMP-2, MMP-9, TIMP-1, and TIMP-2 levels after ovariectomy. Treatment of OVX rats with 1 mg/kg of genistein aglycone significantly restored expression of these proteins whilst lower doses of genistein were shown to increase collagen layer thickness and skin breaking strength [89]. Furthermore, genistein was shown to be able to restore VEGF and TGF-1β, while tissue transglutaminase 2 (TG2) expression decreased due to estrogen deficiency. Intriguingly, genistein (at lower tested concentrations) was able to exert a greater effect than 17-α-ethinyl oestradiol [90].

It was also demonstrated that genistein can impact angiogenesis by affecting migration and adhesion of endothelial cells. Impairment of cell–cell adhesion was observed after treatment of HUVECs with 10 µM concentration of genistein. Limited adhesion was accompanied by down-regulation of mRNA and the protein expression of cell adhesion related genes including VE-cadherin, gap junction protein alpha 1 (connexin 43), integrin alpha V, and multimerin [91]. Furthermore, genistein treatment affected expression of additional proteins linked to adhesion and pro-inflammatory proteins—monocyte chemoattractant protein 1 (MCP-1) and ICAM-1 in brain microvascular endothelial cells (BMECs) [92]. Interestingly, genistein (0.1–5 µM) was shown to inhibit TNF-α-induced endothelial inflammation by decreasing production of adhesion molecules and chemokines such as sICAM-1, sVCAM-1, sE-selectin, MCP-1, and IL-8 [93]. Additionally, genistein also known as a non-selective tyrosine kinase inhibitor is able to decrease migration of endothelial cells by disrupting FAK/paxillin signaling [94].

### 5.3. Epidermis Regeneration

Wound re-epithelization is a crucial process in rebuilding the mechanical barrier between the outer and inner environments. Estrogen also imparts a potent mitogenic effect on keratinocytes, promoting in vitro and in vivo migration [95], which might be affected by the estrogen-mediated ER-β interaction with keratinocytes. ER-β led to an increase in cell proliferation and keratin-19 expression, as well as a decrease in galectin-1 expression. Fittingly, in rat wounds treated with the ER-β agonist, epidermal regeneration was accelerated. In the present study, we provide information on the nuclear ER-β through which selective estrogen receptor agonists affect the expression pattern of selected markers, thus modulating keratinocyte proliferation (increased Ki67 expression) and differentiation (increased keratin 19 expression) [96]. In addition, we demonstrated that the pharmacological activation of ER-α and -β has a different impact on wound healing [96,97].

Genistein among other phytoestrogen-containing extracts modulated the differentiation pattern of keratinocytes (HaCaT) and estrogen positive breast cancer cells (MCF-7). Interestingly, phytoestrogen treatment was shown to increase the expression of luminal marker keratin-8 in MCF-7, but not in HaCaT cells, whereas the expression of other keratins (i.e., 14 and 19) remained rather stable in both used cell lines [98]. Furthermore, estrogen accelerated skin wound healing by promoting keratinocyte proliferation via non-genomic ERK/Akt signaling [99]. Genistein was shown to stimulate hyaluronic acid production in keratinocytes culture in vitro and in vivo [100].

## 6. Maturation and Remodeling Phase

It is well known that abnormalities in wound repair range from non-healing wounds to excessive fibrosis and scarring. Excessive deposition of ECM components and collagens during the maturation/remodeling phase of wound healing may lead to development of hypertrophic scars or keloids. In comparison to hypertrophic scars, which do not grow beyond the boundaries of the wound site, keloids often grow beyond the original extent of the wound. However, both hypertrophic scars and keloids may cause physiological and psychological discomfort [101]. Hypertrophic scar, a more common form of excessive scarring, is often a result of surgical procedures, trauma, radiation, or burn injuries. Although, the exact mechanism of keloid and hypertrophic scar development is not known, various cytokines have been shown to be implicated including IL-6, IL-8, IL-10, and TGF-β as well as various growth factors including for example PDGF [101]. The effect of genistein on maturation phase of wound healing is shown in Figure 2.

In hypertrophic scars, genistein at high concentration (100 µM) was shown to be capable of arresting fibroblast proliferation and suppressing collagen production. On the contrary, normal skin fibroblasts were not affected, which indicates action specificity. In detail, the anti-proliferative effect of genistein was mediated through inhibition of Rat sarcoma (Ras), Rapidly accelerated fibrosarcoma (Raf), ERK, and p38 proteins involved in the MAPK/ERK signaling pathway [28]. Furthermore, in rat cardiac fibroblasts, genistein was shown to inhibit proliferation through GPR30 signaling by suppression of the cell cycle proteins, cyclin B1, and cyclin-dependent kinase 1(CDK1) [27].

Abnormal scarring can also result in keloid formation, characterized by imbalanced ECM synthesis and degradation. One of the features observed in keloids is abnormal activity of transcription factor AP-1 (activator protein 1) connected with disturbed expression of its subunits (c-Fos, c-Jun). In cooperation with other transcription factors, AP-1 induces expression of MMPs and regulates expression of growth factors and cytokines [102]. The expression of C-JUN was higher in keratinocytes treated with both tested concentrations of genistein (37 and 370 µM). On the other hand, in fibroblasts, genistein regulated C-JUN expression in a dose-dependent manner. In normal dermal fibroblasts a lower tested concentration of genistein resulted in higher C-JUN expression whereas in keloid fibroblasts C-JUN expression was more increased at the higher tested concentration of genistein. Genistein at higher tested concentration inhibited C-FOS expression in normal fibroblasts, but the expression of C-FOS was stimulated in keloid fibroblasts in a concentration dependent manner. Of note, genistein, at 370 and 37 µM, inhibited FOS-B expression in keratinocytes [103].

When compared to normal fibroblasts, keloid fibroblasts express higher amounts of connective tissue growth factor (CTGF) on mRNA and protein levels. Genistein was shown to be able to decrease CTGF expression on both levels in a dose dependent manner. Moreover, genistein was also capable of attenuating TGFβ1, β2, and β3 gene expression. Interestingly, the cytoprotective effect of genistein was revealed due to Bcl-2 gene expression stimulation [104]. Furthermore, it was also described in HMVEC-d cells, that the genistein induced Bcl-2 production is mediated rather via ER-β [60].

## 7. Discussion and Conclusions

Better understanding of the biological processes following genistein treatment are inevitable for potential/novel phytoestrogen-based pharmacological manipulations of wound repair. The present data summarize the effects of genistein (Table 1) as natural SERM that has the unique ability to selectively act as agonist or antagonist in a tissue-specific manner. Therefore, genistein improves skin repair [47,90] and simultaneously exerts anti-cancer [105] and chemopreventive properties [106]. As a result several clinical trials combining genistein and/or its analogues with conventional chemotherapeutics have been conducted and point to the safety and efficiency of this drug [107,108]. Nevertheless, further research, including the discovery of the exact underlying mechanisms and optimal wound-type specific management protocol, needs to be performed so that these findings may be applied in practice. Therefore, translation of the reviewed phytoestrogen and estrogen-like compound-mediated signaling to a clinical setting remains a challenge for further research.

## Figures and Tables

**Table 1:** Genistein activity on wound healing.

Phase of Wound Healing	Target Cell	Effect	Reference
Inflammation	Macrophage	↓ activation of NF-κB, STAT-1, iNOS, AMPK	[43,48,49]
		↑ expression of NLRP3	[45,46]
		↓ expression of TNF-α, NF-κB, Il6, IL-1β	[46,47,48]
		M1 → M2 shift promotion	[48]
	T cell,	↓ infiltration	[53]
	Granulocyte	↓ infiltration	[54]
	HUVEC	↓ expression of NF-κB, TNF-α, E-selectin, P-selectin, MCP-1, IL-1β, IL-8, VCAM-1, ICAM-1	[55,56]
	HDMEC	↑ activity of SOD	[59,60]
		↑ expression of Bcl-2	[59,60]
	Fibroblast	↑ levels of GSH	[58]
		↓ expression of MMP-1, MMP-9	[58]
		↓ release of NO, ROS	[58]
	Keratinocyte	↓ expression of TNF-α, IL-6, IL-23, MMP-1, MMP-9	[57,58]
		↑ levels of GSH	[58]
		↓ release of NO, ROS	[58]
Proliferation	Fibroblast	↑/↓ (low/high c) production of collagen	[74]
		↓ activity and production of uPA	[76]
		↓ activation of JNK, ERK1/2	[76]
	HUVEC	↓ proliferation	[83]
		↓ expression of VEGF, VEGFR	[83,84]
		↑ signaling of MAPK	[33]
		↓ signaling of PTK	[33]
		↓ activation of c-Jun, JNK, p38	[33,103]
		↓ production/activation of MMP-2, MMP-9	[33,84]
		↓ VEFG/bFGF-induced expression of MMP-1, uPA and activation of MMP-2	[85,86]
		↑/↓ (low/high dose) stimulation of sprouting	[62]
		↑ (low dose) secretion of bFGF, EGF, angiogenin, angiopoietin-2, MMP-9 and uPA receptor	[88]
		↓ cell-cell adhesion, migration	[91,94]
		↓ expression of VE-cadherin, connexin 43, integrin αV, multimerin	[91]
		↓ TNF-α induced expression of MCP-1, IL-8, sICAM-1, sVCAM-1, E-selectin	[93]
		↓ signaling of FAK/paxillin	[94]
		↑ restores expression of VEGF, TGF-β1, TG2 in estrogen deficiency	[90]
	BMEC	↓ expression of MCP-1, ICAM-1	[92]
	Keratinocyte	↑ proliferation	[99]
		↑ signaling of ERk/Akt	[99]
		↑ production of hyaluronic acid	[100]
		↓ expression of FOS-B	[103]
		↑ expression of Bcl-2	[60,104]
Maturation	Fibroblast	↓ proliferation/collagen production in hypertrophic scars	[28]
		↓ signaling of MAPK/ERK in hypertrophic scars	[28]
		↑ expression of C-JUN, C-FOS in keloid fibroblasts	[103]
		↓ expression of CTGF, TGF-β(1, 2, 3) in keloid fibroblasts	[104]

↑ increase; ↓ decrease.

## Data Availability

Adult human dermal microvascular endothelial cells (HMVEC-d) were obtained from Lonza (Lonza Walkersville, Inc., Walkersville, MD, USA). Detailed description of used methods can be provided by authors on request.

## Abbreviations

AMPK	Adenosine monophosphate-activated protein kinase
AP-1	Activator protein 1
BCRP	Breast cancer resistance protein
bFGF	Basic fibroblast growth factor
Bcl-2	B-cell lymphoma 2
BMECs	Brain microvascular endothelial cells
CCL	C-C motif chemokine
CD	Cluster of differentiation
COL18A1	Collagen Type XVIII Alpha 1 Chain
CTGF	Connective tissue growth factor
CXCL	C-X-C motif chemokine ligand
ECM	Extracellular matrix
EGF	Epidermal growth factor
eNOS	Endothelial nitric oxide synthase
ER	Estrogen receptor
ERK	Signal-regulated kinase
ERT	Estrogen replacement therapy
FoxO1	Orkhead box O transcription factor 1
GPR30	G protein-coupled receptor 30
GSH	Glutathione
GT	Granulation tissue
HaCaT	Human keratinocyte cell line
HMVEC-d	Human dermal microvascular vein endothelial cells
HUVEC	Human umbilical vein endothelial cell
ICAM-1	Intercellular adhesion molecule 1
IGF	Insulin growth factor
IL	Interleukin
iNOS	Inducible nitric oxide synthase
ITGB3	Integrin β3
JAK	Januse kinase
LPC	Lysophosphatidylcholine
LPS	Lipopolysaccharide
MAPK	Mitogen-activated protein kinase
MCF-7	Michigan Cancer Foundation-7
MCP-1	Monocyte chemoattractant protein-1
MMP	Matrix metalloproteinase
MyD88	Myeloid differentiation factor 88
NF-κB	Nuclear factor kappa-light-chain-enhancer of activated B cells
NLRP3	NOD-like receptor protein-3
OVX	Ovariectomized
ox-LDL	Oxidized low density lipoprotein
PDGF	Platelet-derived growth factor
PTK	Protein tyrosine kinase
ROS	Reactive oxygen species
SMA	Smooth muscle actin
SMAD	Sma and MAD-related protein 3
SERM	Selective estrogen receptor modulator
STAT-1	Signal transducer and activator of transcription 1
TIMP	Tissue inhibitor of matrix metalloproteinase
TG2	Transglutaminase 2
TGF	Transforming growth factor
TNF	Tumor necrosis factor
*t*-BHP	*t*-Butylhydroperoxide
uPA	Urokinase-type plasminogen activator
VEGF	Vascular endothelial growth factor
VEGFR	Vascular endothelial growth factor receptor
